# A Study of Short-Chain Fatty Acids During the Canalicular and Early Saccular Phases of Fetal Lung Development and Childhood Asthma

**DOI:** 10.3390/genes15121595

**Published:** 2024-12-13

**Authors:** Huang Lin, Neil J. Perkins, Flory Nkoy, Joseph B. Stanford, Karen C. Schliep, Shyamal D. Peddada

**Affiliations:** 1 Department of Epidemiology and Biostatistics, University of Maryland, College Park, MD 20742, USA; hlin1239@umd.edu; 2 Biostatistics and Bioinformatics Branch (BBB), Eunice Kennedy Shriver National Institute of Child Health and Human Development (NICHD), NIH, Bethesda, MD 20817, USA; perkinsn@mail.nih.gov; 3 Department of Pediatrics, School of Medicine, University of Utah, Salt Lake City, UT 84112, USA; flory.nkoy@hsc.utah.edu; 4 Division of Public Health, School of Medicine, University of Utah, Salt Lake City, UT 84112, USA; joseph.stanford@utah.edu (J.B.S.); karen.schliep@hsc.utah.edu (K.C.S.); 5 Biostatistics and Computational Biology Branch (BCBB), National Institute of Environmental Health Sciences (NIEHS), NIH, Durham, NC 27709, USA

**Keywords:** acetate, asthma, canalicular phase, histone deacetylases (HDACs), fetal immunoprogramming, fetal lungs, saccular phase

## Abstract

Background: Emerging literature indicates that the microbiome and its byproducts, such as short-chain fatty acids (SCFAs), play an important role in childhood diseases such as allergies and asthma. Specifically, there is evidence suggesting that SCFAs play a critical role in fetal immunoprogramming during the late saccular phase of fetal lung development. An increase in acetate during the late saccular phase is known to play a critical role in inhibiting histone deacetylases (HDACs), resulting in a cascade of events, including Treg immune regulation, involved in fetal immunoprogramming, and reduction in the asthma phenotype. However, it is not known whether changes in SCFA levels, especially acetate, occurred during the canalicular or early saccular phase among pregnant women whose children did not develop asthma. Methods: In this research, we investigated this question using plasma samples obtained from mothers during the 20th and 28th weeks of pregnancy. Mothers whose children developed asthma were categorized as cases, while those whose children did not were categorized as controls. The specimens were assayed for a panel of SCFAs consisting of acetate, propionate, butyrate, valerate, isobutyrate, and isovalerate. Results: The resulting data indicated no significant differences between the cases and controls, either at week 20 or week 28, in any of the SCFAs measured, despite the vascularization during these phases. Conclusions: We did not find differences in measured SCFAs at week 20 or at week 28. A larger prospective study covering multiple time points is necessary to confirm the findings of this preliminary study. Such a study, together with the published literature regarding later time points, may help discover critical windows during pregnancy when simple manipulation of diet will result in healthier outcomes for infants.

## 1. Introduction

Childhood asthma and allergies are a worldwide problem, especially in the United States [1,2]. Several factors are potentially associated with childhood asthma such as family history, race, environmental exposures to pollutants, maternal exposures during pregnancy, and others [3]. It is well documented in the literature [4,5,6] that the microbiome and its byproducts, such as short-chain fatty acids (SCFAs), have an effect on immune response and inflammation, and hence human health and disease. This is particularly true in the case of asthma. In the general population, there is greater gut microbial diversity among people without asthma compared to those diagnosed with asthma. There is also a greater abundance of anti-inflammatory taxa and short-chain fatty acids (SCFAs), such as acetate and butyrate, in people without asthma [7].

Understanding the role of gut microbiome and SCFAs in child allergies, atopy, wheeze, and childhood asthma has been an active area of research during the past couple of decades [8,9]. For instance, using stool samples collected three months after birth, Arrieta et al. [8] discovered a significant decrease in the relative abundances of some well-known genera, namely, *Lachnospira, Veillonella, Faecalibacterium*, and *Rothia*, among children with atopy and wheeze, predictors of childhood asthma, compared to healthy controls. Significant reductions in the relative abundances of *Lachnospira* and *Veillonelila* persisted after the first year of life but not for the next two. Furthermore, based on the stool samples collected three months after birth, they also discovered a significant reduction in fecal acetate and no significant reduction in fecal propionate. In view of the changes in the relative abundance of the above genera, the findings regarding acetate are not entirely surprising. Importantly, some of the above-mentioned differentially abundant genera have a role in the production of SCFAs. For example, many strains of *Lachnospira* are involved in acetate production, and *Veillonella* is involved in the production of propionate. *Faecalibacterium*, while consuming acetate, produces butyrate. Several studies during the past decade have demonstrated that childhood atopy, wheeze, and asthma are developmental origin diseases influenced by maternal gut microbiome and SCFAs, specifically acetate, during pregnancy [10,11,12]. Using plasma samples collected during the mid to late third trimester, with median gestational ages of 37.6 weeks (STUDY 1) and 36 weeks (STUDY 2), Thorburn et al. [10] discovered that maternal acetate levels equal to or above the median were associated with a significant decrease in percentage of children requiring two or more general practitioner (GP) visits for cough or wheeze and a trend toward reduced parent-reported wheeze. They reproduced these findings in allergic airway disease (AAD) mice, a rodent model for asthma in humans. Specifically, they showed that the maternal intake of high fiber or acetate during pregnancy was protective for AAD in the adult offspring [10]. They provided important mechanistic insights extending their previous findings that SCFAs, in particular acetate, inhibit histone deacetylases (HDACs) and thus play an important role in the suppression of asthma [10]. Specifically, acetate in blood crosses the placental barrier and inhibits HDACs, in particular HDAC9, resulting in a cascade of events including Treg immune regulation and the suppression of AAD. In a more recent study, Lee-Sarwar et al. [12] investigated the stool SCFA levels during the third trimester of pregnancy of mothers of children who were diagnosed for asthma at about six years of age. They concluded that children born to mothers with higher stool concentrations of acetate relative to other SCFAs during the third trimester are less likely to have atopic asthma compared with children born to mothers with lower acetate concentrations.

It is therefore clear from some of the existing literature that maternal SCFAs during pregnancy, in particular acetate, have a prominent role in childhood atopy, wheeze, and asthma. As noted above, the previous works focused on the third trimester, most likely the mid to late saccular phase of lung development. It is well known that the microbiome and consequently, the concentrations of SCFAs, change during the course of pregnancy. Furthermore, the fetal lungs grow and develop throughout pregnancy. Importantly, the first air-blood barrier is formed with vascularization during the canalicular phase, which occurs between 16 and 24 weeks of gestation [13]. The SCFAs that make their way into the fetal environment through the blood occupy the developing lungs during the canalicular phase. Thus, we hypothesize that this may be one of the earlier windows for fetal immunoprogramming. Since the pre-term babies are subject to a higher risk of developing asthma in childhood [14] and the role of SCFAs, particularly acetate, during early phases of fetal lung development has not been explored in the literature, in this article we studied SCFA concentrations during weeks 20 (mid canalicular phase) and 28 (early saccular phase) of pregnancy. Results of this study together with previous literature corresponding to the mid to late saccular phase of fetal lung development provide a time-course description of the role of maternal SCFAs during pregnancy on childhood asthma.

## 2. Materials and Methods

### 2.1. Study Design

The Effects of Aspirin on Gestation and Reproduction (EAGeR) trial was a prospective, double-blind, placebo-controlled, block-randomized trial (2007–2011) to evaluate the impact of daily preconceptional low dose aspirin (LDA) treatment among women who have experienced one or two pregnancy losses compared to placebo [15]. In an EAGeR follow-up study (2020–2021) [16], we identified 65 children with maternal self-reported wheezing, of whom 38 reported an asthma diagnosis, per a validated questionnaire [17]. An equal number of control children with no report of wheezing or asthma, matched by age, socioeconomic status, smoking status, and sex, were randomly selected from the EAGeR trial for comparison. Among these, children with wheezing/asthma were born to 64 mothers (cases), while children with no wheezing/asthma were born to 63 mothers (controls), with one set of twins. A total of 254 serum samples from the mothers, collected at two time points during pregnancy (20 weeks and 28 weeks of gestational age, 127 samples per time point), were submitted to Creative Proteomics for SCFA sequencing. Following the data quality control process, data from 124 samples at 20 weeks and 127 samples at 28 weeks were used for analysis, with 3 samples at 20 weeks excluded due to low data quality. The SCFA panel consisted of six detectable short-chain fatty acids (See Table A1 in Appendix A).

The original EAGeR trial was approved by the Institutional Review Board (IRB) at each site (Utah site IRB # 00021732) and the Data Coordinating Center in February 2007. All participants (*N* = 1228) gave written informed consent and completed a baseline visit before randomization. University of Utah IRB approval to re-contact EAGeR participants was received before study implementation in November 2019 (UU IRB # 00125444) and remains active. EAGeR participants from the Utah site who provided informed consent were enrolled in the follow-up study, which included completing a questionnaire on their reproductive, cardio-metabolic, and offspring respiratory health 9–14 years after original enrollment.

### 2.2. Statistical Analysis Plan

The primary variable of interest is any reported incidence of wheezing/asthma since birth (Case/Control) in children and we aim to discern the association between maternal SCFA levels during pregnancy and the child’s disease status. All analyses are adjusted for maternal preconception characteristics, including BMI, age, income, sex of the first fetus, and education. Unless noted otherwise, *p*-values are obtained through linear regression. Multiple testing corrections were not implemented because this is a small pilot study which may serve to generate a hypothesis for a future larger prospective study. Missing values in SCFA concentrations are replaced by half of the corresponding LOQ. Since we are primarily interested in describing the association between the levels of individual SCFAs during pregnancy and the disease status of the child, we used the following model for performing the association analysis:

SCFAs∼disease status+BMI+age+income+sex of the first fetus+education.

We further divide the primary analysis into the following sub-analyses:

(1) Assessing whether total SCFA concentrations differ by disease status, stratified by gestational age (20 weeks and 28 weeks).
(2) Evaluating whether the Shannon index of the SCFA profile varies by disease status, stratified by gestational age.
(3) Investigating whether individual SCFAs differ by disease status, stratified by gestational age.
(4) Examining the temporal change (28 weeks–20 weeks) of each individual SCFA by disease status.

In addition to SCFA concentrations, we are also interested in investigating potential changes in SCFA interactions due to the disease status. Thus, as a secondary analysis, we examine the difference-in-differences of Pearson correlation coefficients in SCFAs, specifically assessing whether changes in Pearson correlations over time differ between disease statuses. Permutation tests are employed to determine the statistical significance of these changes.

Sensitivity analyses are also performed to assess the robustness of the conclusions in the primary analysis, including the following:

(1) model without adjusting for confounders: SCFAs∼outcome;
(2) model with centered log-ratio (CLR)-transformed SCFAs without adjusting for confounders: clr-SCFAs∼outcome.

## 3. Results

The baseline socio-demographic characteristics of the mothers included in this study at baseline are detailed in Table 1. We assessed the abundance of each SCFA in the data. All SCFAs, except hexanoate, showed abundances above the limit of quantitation (LOQ). Hexanoate was detected in only one sample and was thus removed from downstream analysis. The summary of SCFA abundance is provided in Table A1.

### 3.1. Primary Analysis

**Figure 1:**
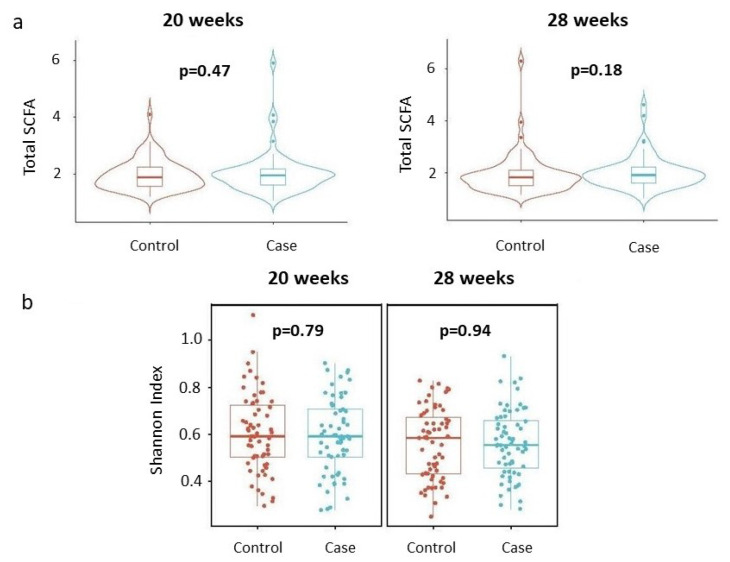
Total concentrations and alpha diversities of SCFAs by disease status, stratified by gestational age. (**a**) Total concentrations. Violin plots describe distributions of total SCFA concentrations (y-axis) across disease status (x-axis). Each violin represents the kernel density estimate of total SCFA concentrations, with the median and interquartile range indicated. Two-sided *p*-values from linear regressions, adjusted for socio-demographic features as detailed in the Section 2, are overlayed in the plot. (**b**) Shannon index. Box plots detail distributions of Shannon index (y-axis) across disease status (x-axis). Each box signifies the interquartile range (IQR) of the data, the median is indicated by the interior line, and whiskers extend to the maximum and minimum values within 1.5 times the IQR from the box. Potential outliers are represented as points outside the whiskers, and jittered points indicate individual data points. Two-sided *p*-values from linear regressions, adjusted for socio-demographic features as detailed in Section 2, are overlayed in the plot. Cases are shown in blue, while controls are shown in red.

We first examined whether maternal total SCFA concentrations and alpha diversities (Shannon index) of the SCFAs differed by the child’s disease status at each time point. The results are summarized in Figure 1a and Figure 1b, respectively. As shown in Figure 1, neither the total SCFA concentrations nor the alpha diversities of SCFAs were significantly different between cases and controls at the two time points considered.

**Figure 2:**
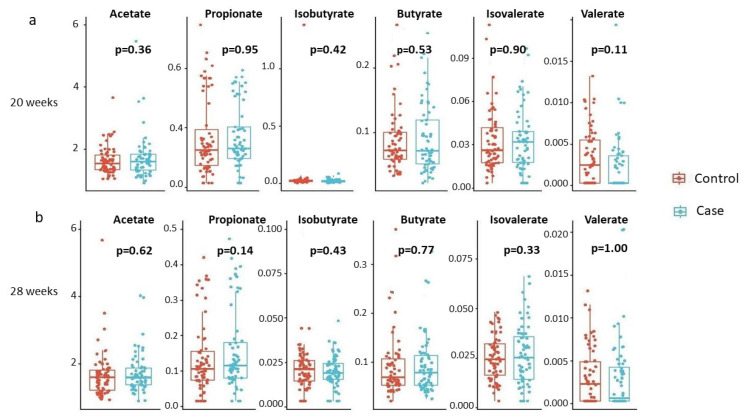
Individual SCFA concentrations by disease status, stratified by gestational age. (**a**) 20 weeks, (**b**) 28 weeks. Box plots detail distributions of each individual SCFA concentration (y-axis) across disease status (x-axis). Each box signifies the interquartile range (IQR) of the data, the median is indicated by the interior line, and whiskers extend to the maximum and minimum values within 1.5 times the IQR from the box. Potential outliers are represented as points outside the whiskers, and jittered points indicate individual data points. Two-sided *p*-values from linear regressions, adjusted for socio-demographic features as detailed in Section 2, are overlayed in the plot. Cases are shown in blue, while controls are shown in red.

We further examined whether individual SCFA concentrations differed by the child’s disease status at each time point. The results are summarized in Figure 2. None of the individual SCFAs showed a statistically significant difference in mean concentrations based on the child’s disease status.

**Figure 3:**
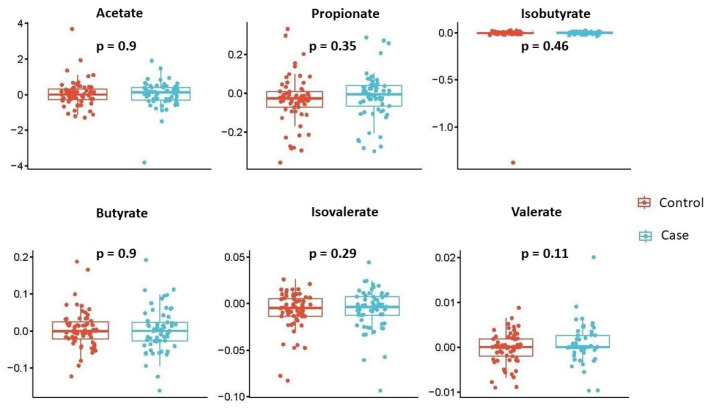
Temporal changes of individual SCFA concentrations by disease status. Box plots describe changes (28 weeks minus 20 weeks) of each individual SCFA concentration (y-axis) across disease status (x-axis). Each box signifies the interquartile range (IQR) of the data, the median is indicated by the interior line, and whiskers extend to the maximum and minimum values within 1.5 times the IQR from the box. Potential outliers are represented as points outside the whiskers, and jittered points indicate individual data points. Two-sided *p*-values from linear regressions, adjusted for socio-demographic features as detailed in Section 2, are overlayed in the plot. Cases are shown in blue, while controls are shown in red.

We also investigated the temporal changes in SCFA concentrations within each subject by computing the difference between 28 weeks and 20 weeks, and examined whether these changes were significantly different based on the child’s disease status. Thus, we compared the two groups of children, namely, cases and controls, in terms of the change in mean SCFA concentrations at week 28 from week 20. The results are summarized in Figure 3. Once again, none of the SCFAs showed statistically significant results, although there was a slightly larger increase in valerate concentrations from week 20 to week 28 in mothers with wheezing/asthmatic children compared to mothers of the control children (*p* = 0.11); however, due to its low abundance, this finding should be interpreted with caution.

### 3.2. Secondary Analysis

**Figure 4:**
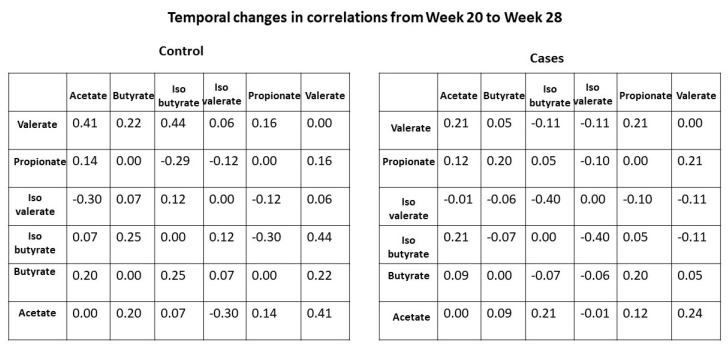
Temporal changes of Pearson correlation coefficients between SCFAs by disease status. Each cell represents the change in correlation coefficient (28 weeks minus 20 weeks) for a specific SCFA pair.

We further examined the temporal changes (28 weeks–20 weeks) in interactions between SCFAs in mothers based on their child’s disease status. As shown in Figure 4, the changes in the Pearson correlation coefficient between isovalerate and isobutyrate are marginally significant between cases and controls (colored in green in Figure 4). Specifically, the correlation between these two SCFAs increased over time in mothers of the control group, while it decreased over time in mothers of the case group.

### 3.3. Sensitivity Analysis

We performed unadjusted analyses on SCFA concentrations both on the original scale and using CLR-transformed values to assess the robustness of our results. The outcomes of these sensitivity analyses closely align with those from the primary analysis, demonstrating the robustness of our findings.

## 4. Discussion

In addition to undergoing hormonal, physiological, and biochemical changes during pregnancy, a woman experiences major shifts temporally in the composition of the gut and vaginal microbiome as she prepares for childbirth and lactation. These lead to changes in the composition of microbial byproducts such as SCFAs and various metabolites, which have critical functions during the course of pregnancy. While the microbes themselves may not enter the fetal environment, the microbial byproducts, specifically the SCFAs, do enter the fetal environment as they have multiple roles, including fetal immunoprogramming. Specifically, acetate enters the fetal environment to inhibit histone deacetylases (HDACs). Histones play a critical role during the cell division cycle by tightly winding the chromatin to maintain the integrity of chromosomes during the duplication phase. However, for fetal immunoprogramming, it is necessary to inhibit HDACs for the transcription of Foxp3 and the expression of several genes such as Nppa in the fetal lung, which have a critical role in immune regulation and Treg biology [10]. Acetate plays a critical role in inhibiting HDACs. Since acetate is produced by bacteria acting on undigested dietary fiber, it is therefore important for a pregnant woman to consume a high fiber diet during pregnancy to increase the production of acetate. A question of interest is whether there is a critical window of fetal development when high levels of acetate are required for the offspring of a pregnant woman to benefit from her consumption of a higher fiber diet. Existing literature indicates that high levels of acetate around the 37th week of pregnancy protects against childhood allergies and asthma. In addition to investigating the differences in mean SCFA levels between cases and controls, we also explored changes in the pairwise correlations between pairs of SCFAs from week 20 to week 28 in the cases and controls. Although we did not see any differences in the mean SCFA levels, we discovered that the correlations between isovalerate and isobutyrate increased between the two time points in mothers of the control group, while it decreased between the two time points in mothers of the case group. Although this finding is marginally statistically significant, it suggests a possible differential interaction between these two short-chain fatty acids in cases and controls.

This study has several limitations that should be considered when interpreting the findings. First, important confounders such as maternal diet, environmental factors, and social determinants of health—including the Childhood Opportunity Index (COI) and Areas of Deprivation Index (ADI)—were not controlled for. Second, the study did not account for the age of asthma onset but focused on whether or not the subjects had asthma. Hence, the observed associations need to be interpreted accordingly. Third, the binary classification of asthma into case/control groups oversimplifies the condition because asthma exists on a spectrum with varying severity and phenotypes. Furthermore, while SCFA levels were analyzed at gestational weeks 20 and 28, other potentially critical developmental windows, such as those earlier in pregnancy or between weeks 28 and 40, were not explored. While surveying the entire gestational period would be ideal, this pilot study provides valuable information that will guide future large-scale studies. Specifically, despite the negative findings, this study is informative for a future follow-up study to discover a critical window of the effects of SCFAs, in particular, acetate. Additionally, the asthma outcomes were based on maternal self-reports collected 9–14 years after the original EAGeR trial, introducing potential recall bias and reducing reliability, as no clinical diagnoses or objective measures were used. It is also important to note that the study population was drawn exclusively from the EAGeR trial, which recruited women with a history of pregnancy loss. This unique population, combined with the single-site focus in Utah, limits the generalization of the findings to broader populations and diverse environments. Finally, the data analysis framework does not allow for modeling causality, restricting the ability to draw definitive conclusions about the relationship between SCFA levels and asthma risk.

Although the study may seem underpowered due to small sample sizes, the effect sizes for almost all SCFAs between groups at both time points are very small. The means of almost all comparison groups are very close to each other. One may require an extremely large sample size to detect such small effect sizes with any reasonable power. Even if one achieves statistical significance to detect such small differences, they may not be biologically relevant. Thus, while this study may be underpowered, given the effect sizes, the biological interpretations are unlikely to change with larger sample sizes at the two time points analyzed.

Our goal was to investigate whether changes in SCFA levels occurred during the canalicular or early saccular phases of lung development in pregnant women whose children later developed asthma. However, in this preliminary investigation, we did not observe significant differences in SCFA levels in either the 20- or 28-week samples. Notably, previous studies have identified signals, particularly changes in acetate levels, around 36 and 37.6 weeks of gestation. While our findings are negative, they help to refine the timeline by narrowing the potential critical window for SCFA-related effects to the period between 28 and 36 weeks of gestation. This insight highlights the need for large-scale, prospective dose-response studies to examine the effects of dietary fiber intake during this critical window on SCFA concentrations and the risk of childhood allergies or asthma. Such studies could pave the way for identifying non-invasive biomarkers for early childhood asthma intervention. For example, prior research suggests that supplementation with specific metabolites, such as SCFAs, holds the potential for modulating immune responses and reducing inflammation [18,19], which may inform maternal nutrition and prenatal care practices.

## Figures and Tables

**Table 1:** Summary of maternal socio-demographic characteristics at baseline.

	Control (N=63)	Case (N=64)	Total (N=127)
BMI			
min	15.71	15.81	15.71
max	45.9	42.89	45.9
mean (sd)	25.68 (5.77)	25.93 (5.75)	25.81 (5.74)
Age			
min	21.18	19.51	19.51
max	37.11	38.39	38.39
mean (sd)	25.68 (5.77)	25.93 (5.75)	25.81 (5.74)
Income (%)			
Annual less than $19,999	2 (3)	2 (3)	4 (3)
$20,000–$39,999	13 (21)	16 (25)	29 (23)
$40,000–$74,999	8 (13)	7 (11)	15 (12)
$75,000–$99,999	10 (16)	5 (8)	15 (12)
$100,000	30 (48)	34 (53)	64 (50)
First fetus sex (%)			
Female	29 (46)	30 (47)	59 (46)
Male	34 (54)	34 (53)	68 (54)
Education (%)			
Not high-school graduate	1 (2)	1 (2)	2 (2)
High-school graduate	3 (5)	8 (12)	11 (9)
>High school	59 (94)	55 (86)	114 (90)

## Data Availability

The research data presented in this study are available upon completion of a data usage agreement (DUA) with Dr. Neil Perkins (perkinsn@mail.nih.gov). All analytical code and computational pipelines used in this study are publicly accessible through our GitHub repository (https://github.com/FrederickHuangLin/Asthma-SCFA-Project (accessed on 7 December 2024)).

## Abbreviations

The following abbreviations are used in this manuscript:

SCFA	Short-chain fatty acid
HDACs	Histone deacetylases
LOQ	Limit of quantitation

## Appendix A

**Table A1:** Summary of SCFA abundance from maternal serum samples.

	20 Weeks (N=124)	28 Weeks (N=127)	Total (N=251)
Acetate			
Above LOQ	124(100)	127(100)	251(100)
Below LOQ	0(0)	0(0)	0(0)
Propionate			
Above LOQ	115 (93)	117(92)	232(92)
Below LOQ	9(7)	10(8)	19(8)
Isobutyrate			
Above LOQ	121 (98)	116(91)	237 (94)
Below LOQ	3(2)	11 (9)	14(6)
Butyrate			
Above LOQ	124(100)	127(100)	251(100)
Below LOQ	0(0)	0(0)	0(0)
Isovalerate			
Above LOQ	119(96)	121(95)	240(96)
Below LOQ	5(4)	6(5)	11(4)
Valerate			
Above LOQ	65(52)	69(54)	134(53)
Below LOQ	59(48)	58(46)	117(47)
Hexanoate			
Above LOQ	1 (1)	0(0)	1(0)
Below LOQ	123 (99)	127(100)	250(100)

## References

[1] Serebrisky D., Wiznia A. (2019). Pediatric asthma: A global epidemic. Ann. Glob. Health.

[2] Pearce N., Aït-Khaled N., Beasley R., Mallol J., Keil U., Mitchell E., Robertson C. (2007). Worldwide trends in the prevalence of asthma symptoms: Phase III of the International Study of Asthma and Allergies in Childhood (ISAAC). Thorax.

[3] Johnson C.C., Havstad S.L., Ownby D.R., Joseph C.L., Sitarik A.R., Myers J.B., Gebretsadik T., Hartert T.V., Hershey G.K.K., Jackson D.J. (2021). Pediatric asthma incidence rates in the United States from 1980 to 2017. J. Allergy Clin. Immunol..

[4] Hacquard S., Garrido-Oter R., González A., Spaepen S., Ackermann G., Lebeis S., McHardy A.C., Dangl J.L., Knight R., Ley R. (2015). Microbiota and host nutrition across plant and animal kingdoms. Cell Host Microbe.

[5] Yang Q., Liang Q., Balakrishnan B., Belobrajdic D.P., Feng Q.J., Zhang W. (2020). Role of dietary nutrients in the modulation of gut microbiota: A narrative review. Nutrients.

[6] Eckburg P.B., Bik E.M., Bernstein C.N., Purdom E., Dethlefsen L., Sargent M., Gill S.R., Nelson K.E., Relman D.A. (2005). Diversity of the human intestinal microbial flora. Science.

[7] Wang Q., Li F., Liang B., Liang Y., Chen S., Mo X., Ju Y., Zhao H., Jia H., Spector T.D. (2018). A metagenome-wide association study of gut microbiota in asthma in UK adults. BMC Microbiol..

[8] Arrieta M.C., Stiemsma L.T., Dimitriu P.A., Thorson L., Russell S., Yurist-Doutsch S., Kuzeljevic B., Gold M.J., Britton H.M., Lefebvre D.L. (2015). Early infancy microbial and metabolic alterations affect risk of childhood asthma. Sci. Transl. Med..

[9] Zhao X., Hu M., Zhou H., Yang Y., Shen S., You Y., Xue Z. (2023). The role of gut microbiome in the complex relationship between respiratory tract infection and asthma. Front. Microbiol..

[10] Thorburn A.N., McKenzie C.I., Shen S., Stanley D., Macia L., Mason L.J., Roberts L.K., Wong C.H., Shim R., Robert R. (2015). Evidence that asthma is a developmental origin disease influenced by maternal diet and bacterial metabolites. Nat. Commun..

[11] Vuillermin P.J., O’Hely M., Collier F., Allen K.J., Tang M.L., Harrison L.C., Carlin J.B., Saffery R., Ranganathan S., Sly P.D. (2020). Maternal carriage of Prevotella during pregnancy associates with protection against food allergy in the offspring. Nat. Commun..

[12] Lee-Sarwar K.A., Kelly R.S., Lasky-Su J., Zeiger R.S., O’Connor G.T., Sandel M.T., Bacharier L.B., Beigelman A., Rifas-Shiman S.L., Carey V.J. (2020). Fecal short-chain fatty acids in pregnancy and offspring asthma and allergic outcomes. J. Allergy Clin. Immunol. Pract..

[13] Schittny J.C. (2017). Development of the lung. Cell Tissue Res..

[14] Caffarelli C., Gracci S., Giannì G., Bernardini R. (2023). Are Babies Born Preterm High-Risk Asthma Candidates?. J. Clin. Med..

[15] Schisterman E.F., Silver R.M., Perkins N.J., Mumford S.L., Whitcomb B.W., Stanford J.B., Lesher L.L., Faraggi D., Wactawski-Wende J., Browne R.W. (2013). A randomised trial to evaluate the effects of low-dose aspirin in gestation and reproduction: Design and baseline characteristics. Paediatr. Perinat. Epidemiol..

[16] Shaaban M., Shepelak Z.D., Stanford J.B., Silver R.M., Mumford S.L., Schisterman E.F., Hinkle S.N., Nkoy F.L., Theilen L., Page J. (2024). Low-dose aspirin, maternal cardiometabolic health, and offspring respiratory health 9 to 14 years after delivery: Findings from the EAGeR Follow-up Study. Paediatr. Perinat. Epidemiol..

[17] Asher M.e., Keil U., Anderson H., Beasley R., Crane J., Martinez F., Mitchell E., Pearce N., Sibbald B., Stewart A. (1995). International Study of Asthma and Allergies in Childhood (ISAAC): Rationale and methods. Eur. Respir. J..

[18] Trompette A., Gollwitzer E.S., Yadava K., Sichelstiel A.K., Sprenger N., Ngom-Bru C., Blanchard C., Junt T., Nicod L.P., Harris N.L. (2014). Gut microbiota metabolism of dietary fiber influences allergic airway disease and hematopoiesis. Nat. Med..

[19] Cait A., Hughes M., Antignano F., Cait J., Dimitriu P., Maas K., Reynolds L., Hacker L., Mohr J., Finlay B. (2018). Microbiome-driven allergic lung inflammation is ameliorated by short-chain fatty acids. Mucosal Immunol..

